# Psychedelics for anxiety disorders: Challenges and opportunities

**DOI:** 10.1016/j.fmre.2025.07.008

**Published:** 2025-07-16

**Authors:** Chenshu Gao, Heming Cheng

**Affiliations:** Zhejiang Collaborative Innovation Center for the Brain Diseases with Integrative Medicine, Zhejiang Key Laboratory of Neuropsychopharmacology, School of Pharmaceutical Sciences, The First Affiliated Hospital, Zhejiang Chinese Medical University, Hangzhou 310053, China

A recent study published in *Neuron* by Tiwari *et al*. identified the critical role of 5-HT_2A_ receptor in ventral hippocampal parvalbumin (PV) interneurons (INs) in mediating the acute anxiolytic effects of the serotonergic psychedelic 2,5-dimethoxy-4-iodoamphetamine (DOI). This study not only advances psychedelic neuropharmacology but also provides a robust framework for developing novel rapid-acting therapeutics for anxiety disorders [1].

Anxiety disorder is one of the most prevalent psychiatric conditions, yet its neurobiological substrates are multifaceted and incompletely understood. Conventional pharmacological interventions, particularly selective serotonin reuptake inhibitors (SSRIs), are limited by their delayed therapeutic onset, typically requiring several weeks to achieve clinical efficacy [2]. Intriguingly, emerging evidence reveals that serotonergic psychedelics demonstrate rapid-onset and sustained anxiolytic properties [3]; nevertheless, systematic investigation into their neural mechanisms remains sparse. The anxiolytic properties of DOI were assessed across multiple behavioral paradigms, including the marble burying test (MBT), four plates test (FPT), elevated plus maze (EPM), and light/dark box (LDB). Acute systemic DOI administration (0.3–2 mg/kg) elicited dose-dependent anxiolytic responses in the MBT, FPT, and EPM, yet failed to demonstrate comparable efficacy in the LDB. Neuroanatomically, divergent outcomes emerged: intrahippocampal microinjection of 5 µg DOI induced anxiolysis in the FPT, while identical doses administered to the amygdala or periaqueductal gray (PAG) paradoxically evoked robust anxiogenic responses [4]. In addition, DOI acts as a partial agonist at 5-HT_2A_ receptors while retaining moderate affinity for 5-HT_2A_ and 5-HT_2C_ subtypes, which likely underlies its complex psychotropic manifestations, ranging from therapeutic anxiolysis to hallucinogenic effects [5]. These region- and receptor-specific dichotomies underscore the necessity to delineate precise neuroanatomical substrates, cellular targets, and receptor subtypes underlying the rapid anxiolytic actions of DOI.

Tiwari *et al*. found that system administration of 1 mg/kg DOI produced acute anxiolysis in the EPM across rodent strains and sexes, with effects diminishing 24 h post-administration. Anxiety-related neural dysfunction arises from complex interactions across brain regions, cell types, and circuits (Fig. 1). Localized DOI infusion (1 µg) into the bilateral ventral CA1/subiculum (vCA1/Sub), but not the medial prefrontal cortex (mPFC), basolateral amygdala (BLA), or dorsal CA1/subiculum, replicated systemic anxiolysis. As a hub of the limbic system, the hippocampus integrates cortical inputs and relays processed signals to downstream regions, governing critical functions including learning, memory, and emotional regulation. Using in vivo electrophysiology and c-Fos staining, Tiwari *et al*. found that DOI preferentially enhances activity in vCA1/sub PV interneurons. Chemogenetic activation of these PV INs mimics the anxiolytic effect of DOI. These findings suggest that DOI likely exerts rapid anxiolytic effects by activating vCA1/sub PV INs. Notably, DOI also increased firing rates in a subset of regular-spiking (RS) glutamatergic pyramidal neurons (PNs), though c-Fos expression in CaMKIIα-positive PNs remained unchanged. Recent studies suggest that vCA1 PV INs exhibit activation in open-arm environments and exert anxiolytic effects by inhibiting anxiety-promoting glutamatergic neurons through local microcircuits [6]. Therefore, there could be distinct populations of glutamate neurons, with one anxiety-promoting population being inhibited by DOI-activated PV INs, while another may be activated by DOI. The microcircuits of PV INs and functional roles of DOI-activated glutamatergic neurons warrant further exploration. Moreover, Tiwari *et al*. recorded neuronal activity under normal conditions, while further studies could investigate the real-time responses of glutamatergic and PV neurons to DOI administration in an anxiety-inducing environment using techniques such as miniaturized two-photon microscopy.

**Fig. 1:**
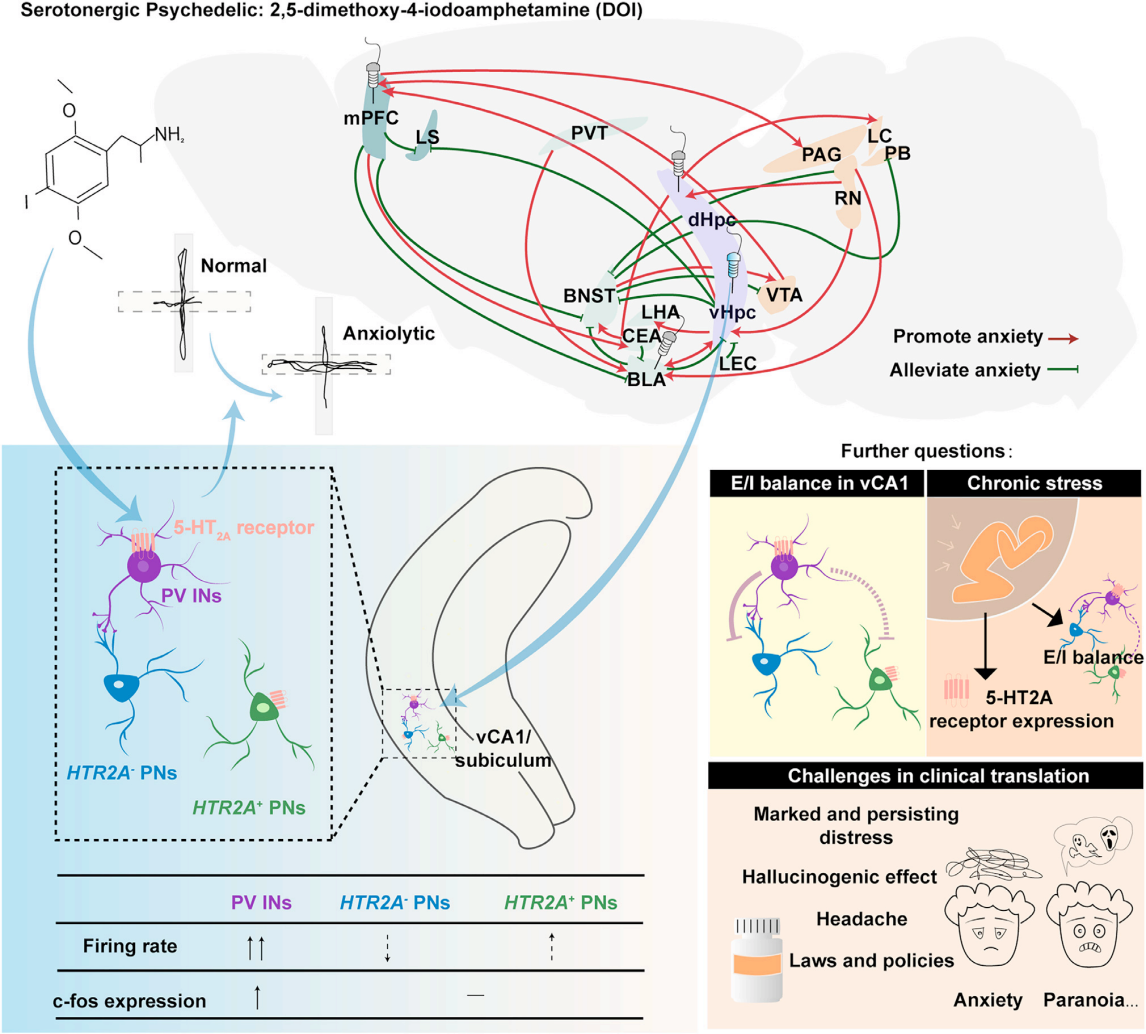
**Neural substrates underlying the anxiolytic effect of the serotonergic psychedelic DOI and further questions and challenges.** The acute anxiolytic actions of the serotonergic psychedelic DOI are mediated by the 5-HT_2A_ receptor on parvalbumin interneurons (PV INs) in the ventral hippocampal CA1/subiculum region. The lower-left table shows that PV INs are activated by DOI, while glutamate neurons may include one anxiety-promoting population being inhibited by DOI-activated PV INs and another activated by DOI. However, several issues remain to be addressed. Question 1: How does DOI regulate the excitatory-inhibitory (E/I) balance in vCA1? Question 2: Are 5-HT_2A_ receptor expression and DOI’s regulation of hippocampal E/I balance altered in anxiety disorders? Question 3: Further challenges for DOI in clinical translation. Abbreviations: mPFC, medial prefrontal cortex; LS, lateral septum; PVT, thalamic paraventricular nucleus; BNST, bed nucleus of the stria terminalis; LHA, lateral hypothalamic area; CEA, central nucleus of the amygdala; BLA, basolateral amygdala; LEC, lateral entorhinal cortex; VTA, ventral tegmental area; vHpc, ventral hippocampus; dHpc, dorsal hippocampus; RN, raphe nucleus; PAG, periaqueductal gray; LC, locus coeruleus; PB, parabrachial nuclei; PNs, pyramidal neurons.

As a partial agonist of 5-HT_2A_ receptors with cross-reactivity at 5-HT_2A_ and 5-HT_2C_ receptors, DOI’s receptor-dependent mechanisms were dissected using pharmacological and genetic tools. Intrahippocampal infusion of a selective 5-HT_2A_ receptor antagonist or global 5-HT_2A_ receptor knockout abolished the anxiolytic effects of DOI. Strikingly, restoring 5-HT_2A_ receptors specifically in PV INs, but not CamKⅡα neurons, rescued the attenuated anxiolytic effect of DOI on 5-HT_2A_RKO mice. These results suggest that the 5-HT_2A_ receptor in the ventral CA1/sub PV INs may serve as a hub for the anxiolytic actions of serotonin psychedelics. Interestingly, despite robust 5HT_2A_ receptor expression on CamKⅡα neurons, their activity remains minimally affected by DOI, potentially due to inhibitory inputs from PV INs. However, more experiments are needed to verify whether DOI acts by binding directly to 5-HT_2A_ receptors or indirectly activating these receptors by enhancing the release of 5-HT in the ventral CA1/sub. Interestingly, nearly 67% of CamKⅡα neurons also exhibit robust 5HT_2A_ receptor expression, and their activity and function can be further investigated by applying CamKIIα promoter CRE-dependent viruses in HTR2A-cre mice, which enables precise manipulation of 5HT_2A_ receptor-expressing PN subpopulations.

Overall, enthusiasm exists regarding the promising effects of psychedelics in treating neuropsychiatric disorders such as anxiety. Although Tiwari *et al*. demonstrate an excellent breakdown of the 5HT_2A_ receptor-mediated PV microcircuit in the ventral hippocampus controlling anxiety, the potential use of DOI for anxiety disorders remains largely unexplored. The hippocampus exhibits explicit vulnerability to chronic anxiety-inducing stimuli [7]. Studies have found that chronic stress leads to a reduction in PV IN numbers and morphological changes. Consequently, an imbalance in hippocampal excitation-inhibition (E/I) is considered a contributing factor to anxiety disorders. Therefore, further research is needed to explore the anxiolytic effects of DOI on this imbalance/pathological state. Additionally, chronic stress can also induce changes in *HTR2A* mRNA levels [8]. Given the widespread expression of 5-HT_2A_ receptors in PV INs and PNs, it is worthwhile to further investigate both the characterization of 5-HT_2A_ receptor expression under stress and the effects of DOI on these two neuronal populations.

Crucially, Tiwari *et al*. identify the 5HT_2A_ receptor in hippocampal PV INs as a promising target for anxiety treatment using DOI. While psychedelic therapies offer therapeutic potential, they carry inherent risks. Although studies indicate that a single administration of psychedelics (e.g., psilocybin) can produce sustained therapeutic effects through mPFC synaptic plasticity [3], Tiwari *et al*. found that the anxiolytic effects of DOI diminished 24 h following acute administration. Notably, they observed that acute vCA1/sub-targeted DOI did not induce the head twitch response (HTR)**—**a behavior hallmark of hallucination, suggesting spatial and mechanistic segregation between therapeutic and psychotomimetic effects [9]. However, the chronic DOI exposure effects require further validation, particularly since repeated administration alters 5-HT_2A_ receptor expression and induces tolerance. Clinically, these treatments may provoke “challenging experiences” (e.g., anxiety, paranoia) [10], necessitating deeper mechanistic insights to optimize safety.

In conclusion, Tiwari *et al*. demonstrated that the 5-HT_2A_ receptors on vCA1/sub PV INs contribute to the acute anxiolytic action evoked by DOI, thereby providing a novel perspective on the neural mechanisms for the rapid anxiolytic effects of psychedelics. This research lays the foundation for future therapies involving structural modification of psychedelics or screening for other 5-HT_2A_ receptor-targeting compounds. Furthermore, novel strategies for cell-type-specific delivery to vCA1/subiculum PV INs could minimize the off-target effects of psychedelics, though technical challenges in achieving this remain a long-term research goal. Potential future approaches include novel vectors (e.g., targeted AAVs), chemogenetic strategies, and nanovesicles, though all remain exploratory and require robust regulatory frameworks to support their medical applications.

## CRediT authorship contribution statement

**Chenshu Gao:** Writing – original draft. **Heming Cheng:** Writing – review & editing, Conceptualization.

## References

[1] Tiwari P., Davoudian P.A., Kapri D. (2024). Ventral hippocampal parvalbumin interneurons gate the acute anxiolytic action of the serotonergic psychedelic DOI. Neuron.

[2] Sartori S.B., Singewald N. (2019). Novel pharmacological targets in drug development for the treatment of anxiety and anxiety-related disorders. Pharmacol Ther.

[3] Agnorelli C., Spriggs M., Godfrey K. (2025). Neuroplasticity and psychedelics: A comprehensive examination of classic and non-classic compounds in pre and clinical models. Neurosci Biobehav Rev.

[4] Werle I., Bertoglio L.J. (2024). Psychedelics: A review of their effects on recalled aversive memories and fear/anxiety expression in rodents. Neurosci Biobehav Rev.

[5] Cameron L.P., Benetatos J., Lewis V. (2023). Beyond the 5-HT(2A) receptor: Classic and nonclassic targets in psychedelic drug action. J Neurosci.

[6] Volitaki E., Forro T., Li K. (2024). Activity of ventral hippocampal parvalbumin interneurons during anxiety. Cell Rep.

[7] Shi H.J., Wang S., Wang X.P. (2023). Hippocampus: Molecular, cellular, and circuit features in anxiety. Neurosci Bull.

[8] Zou L., Tian Y., Wang Y. (2023). High-cholesterol diet promotes depression- and anxiety-like behaviors in mice by impact gut microbe and neuroinflammation. J Affect Disord.

[9] Muir J., Lin S., Aarrestad I.K. (2024). Isolation of psychedelic-responsive neurons underlying anxiolytic behavioral states. Science.

[10] Barrett F.S., Bradstreet M.P., Leoutsakos J.S. (2016). The Challenging Experience Questionnaire: Characterization of challenging experiences with psilocybin mushrooms. J. Psychopharmacol. (Oxford).

